# Paratesticular Fibrous Pseudotumor: Averting Radical Orchiectomy

**DOI:** 10.7759/cureus.72432

**Published:** 2024-10-26

**Authors:** Eros Qama, Shamsu Bello, Garrison Pease

**Affiliations:** 1 Pathology and Laboratory Medicine, Albert Einstein College of Medicine, Bronx, USA

**Keywords:** diagnostic imaging, histopathology, paratesticular fibrous pseudotumor, radical orchiectomy, testis-sparing surgery

## Abstract

Paratesticular fibrous pseudotumors are rare, benign proliferations that pose significant diagnostic challenges due to their resemblance to fibroblastic soft tissue tumors. We report a young adult male who presented with a right scrotal mass, initially considered to be a solitary fibrous tumor (SFT) but ultimately diagnosed as a fibrous pseudotumor. Histopathologic examination revealed a well-circumscribed, hyalinized, hypocellular spindle cell mass with inflammatory features. Despite the marginal Signal transducer and activator of transcription 6 (STAT6) positivity, extensive consultation confirmed the benign nature of the mass. This case highlights the importance of meticulous diagnostic processes, including advanced imaging and intraoperative assessments, to avoid unnecessary orchiectomy. It also highlights the characteristic histologic features that differentiate these benign entities from malignant counterparts, ensuring appropriate management and a favorable prognosis.

## Introduction

First described by Balloch in 1904, paratesticular fibrous pseudotumors are benign proliferations originating from the tunica layers of the testes [1]. A century later, subsequent work by Jones et al. refined the understanding of these entities, pinpointing their origin predominantly within the paratesticular region and accounting for approximately six percent of all paratesticular masses [2]. The fibroinflammatory nature of these lesions, possibly due to trauma, infectious processes, or chronic inflammation, complicates their differential diagnosis, emphasizing the necessity for advanced diagnostic awareness to distinguish them from well-defined fibroblastic tumors [2]. Conventionally, diagnosis commences with ultrasound, despite its specificity constraints, often necessitating adjunctive magnetic resonance imaging (MRI) and definitive histopathologic analysis post-resection [3]. Therapeutically, the emphasis on testis-sparing modalities, particularly partial orchiectomy augmented by intraoperative frozen section analysis, underscores the imperative of fertility preservation concurrent with oncologic integrity [4].

## Case presentation

**Figure 1:**
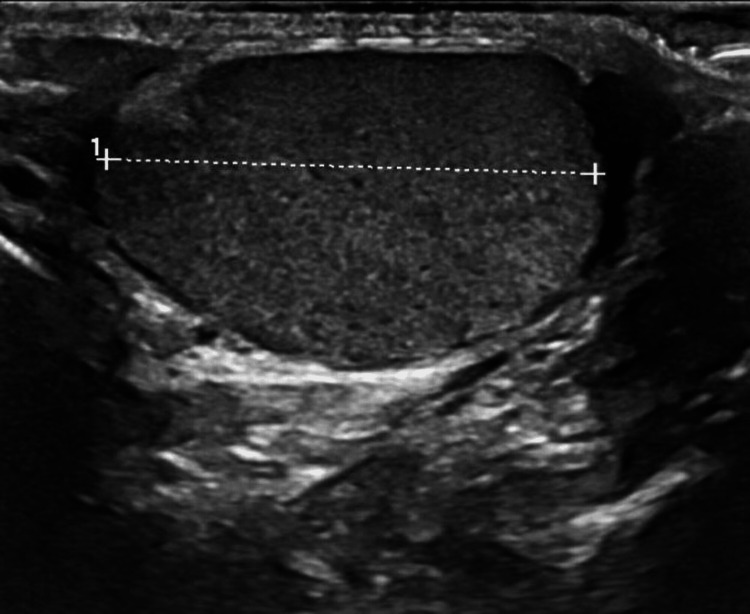
Scrotal ultrasonography revealed an intrascrotal extratesticular mass measuring approximately 3 cm.

A young adult male with an unremarkable medical history presented to the emergency department with a right scrotal mass that had been palpable for several months, accompanied by a brief history of dysuria. On examination, a non-tender, mobile mass was palpated at the superior pole of the right testis without induration, erythema, fluctuance, or exudation. Scrotal ultrasonography revealed an intrascrotal extratesticular mass measuring approximately 3 cm (Figure 1).

**Figure 2:**
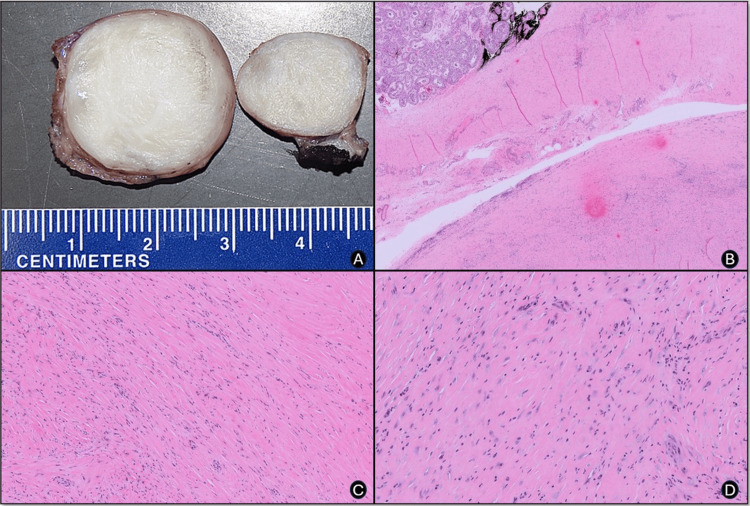
Intraoperative image and H&E H&E: Hematoxylin and eosin A: Intraoperative image showing a well-circumscribed, pedunculated extratesticular mass with a tan-white, rubbery cut surface B: Low-power photomicrograph displaying the extratesticular mass adjacent to normal seminiferous tubules (H&E, original magnification ×20) C: Medium-power view illustrating bundles of fibroblastic proliferation within dense hyalinized fibrous tissue (H&E, original magnification ×40) D: High-power image showing spindle-shaped fibroblasts arranged in fascicles with abundant cytoplasm and large nuclei (H&E, original magnification ×100)

Given the differential diagnosis, which included a torsed testicular appendage or a tumor, surgical exploration was planned for both diagnosis and management. Intraoperatively, a well-demarcated paratesticular mass was identified and excised (Figure 2A). Frozen section analysis identified the mass as a hypocellular spindle cell lesion, raising concern for a potential low-grade sarcoma. Consequently, a radical orchiectomy was performed.

In the postoperative examination, hematoxylin and eosin (H&E)-stained specimens revealed an approximately 3 cm, well-circumscribed, hyalinized, sclerotic, hypocellular spindle cell mass characterized by tapered nuclei and interspersed lymphocytic and plasmacytic inflammation (Figures 2B-2D).

**Figure 3:**
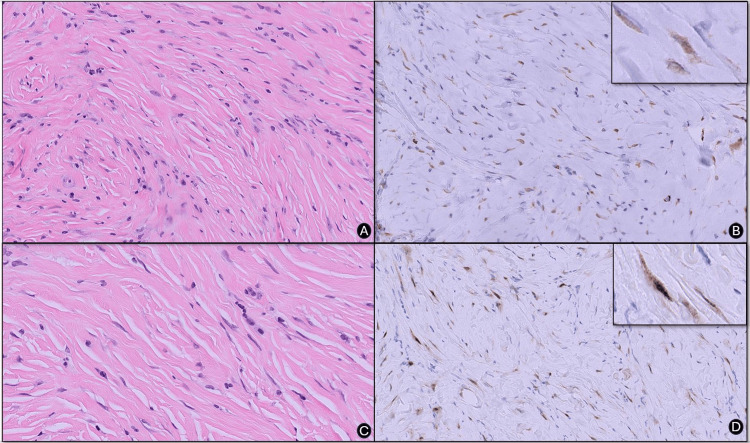
STAT6 STAT6: Signal transducer and activator of transcription 6; H&E: Hematoxylin and eosin A: High-power view of fibroblastic fascicles (H&E, original magnification ×200) B: Initial STAT6 immunostaining showing subtle nuclear positivity in spindle cells (original magnification ×200). Inset: Enlarged view of a fibroblast with marginal STAT6 positivity (×400). C. Bundles of fibroblastic fascicles (H&E, original magnification ×200) D: Consultative STAT6 immunostaining depicting similar findings interpreted as nonspecific (original magnification ×200). Inset: High-power view of spindle cell with marginal staining (×400).

The testicular parenchyma appeared unremarkable, clearly demarcated from, and not infiltrated by the mass. Notably, the mass did not exhibit necrosis, mitotic activity, or cellular atypia (Figure 3A). Initially, the differential diagnosis leaned toward a fibrous pseudotumor, a benign condition. However, given the morphologic similarities between fibrous soft tissue tumors and fibrous pseudotumors particularly the extensive hyalinization, sclerotic changes, and a hypocellular spindle cell mass with tapered nuclei-consultation with additional in-house pathologists were made to consider excluding a solitary fibrous tumor (SFT) using signal transducer and activator of transcription 6 (STAT6) despite the absence of variable cellularity and prominent staghorn vascular structures.

The immunohistochemical (IHC) assessment for STAT6 showed subtle positivity. Although marginal, it suggested some phenotypic convergence with the intraoperative diagnosis of a spindle cell soft tissue tumor (Figures 3B-3C). This initial STAT6 IHC finding led to a preliminary diagnosis of SFT. Upon outside consultation and a repeat STAT6, a subtle positive focal expression was observed again, similar to the in-house results (Figure 3D). However, the external review interpreted this staining as non-specific, leading to the final diagnosis of a paratesticular fibrous pseudotumor.

## Discussion

Fibrous pseudotumors of the testis and paratesticular structures are rare, benign entities characterized by fibrous tissue proliferation [5]. They present as either solitary or multiple nodules and mimic malignant testicular tumors both clinically and radiologically, posing significant diagnostic challenges [6]. Their low incidence leads to rare clinical encounters and a limited number of extensive studies or large case series in the literature [7].

Non-calcifying fibrous pseudotumors, a distinct variant within this category, are characterized by the absence of calcifications, a feature sometimes present in other fibrous pseudotumors. These tumors exhibit a homogeneous fibroblast population within a collagenous stroma, unlike the varied cellular makeup typical of many testicular and paratesticular masses [8]. This consistent cellularity is crucial for distinguishing these tumors from neoplastic processes, which generally display greater cellular heterogeneity [9].

The histopathologic hallmark of fibrous pseudotumors, including non-calcifying types, is their characteristic vascular pattern: orderly, thin-walled vessels embedded within dense fibrous tissue, contrasting sharply with the tortuous or complex networks seen in malignant vascular proliferations [5]. The etiology of these tumors is often elusive, typically linked to a reactive response to trauma, inflammation, or autoimmune reactions that promote localized fibroblastic and myofibroblastic proliferation [6,9]. Clinically, they may present as a palpable scrotal mass, sometimes accompanied by discomfort, necessitating differentiation from malignant testicular tumors [7]. Although ultrasound and MRI are informative, definitive diagnosis relies on histopathologic examination, with surgical excision being the preferred treatment to confirm the diagnosis and resolve the lesion [10].

## Conclusions

The post-excision prognosis for fibrous pseudotumors, including non-calcifying variants, is favorable, with minimal risk of recurrence. This underscores the importance of accurately differentiating these benign tumors from malignancies to avoid unnecessary radical treatments such as orchiectomy. In conclusion, these tumors are key considerations in the differential diagnosis of testicular and paratesticular masses. Recognizing their unique histopathologic features, such as uniform cellularity and distinctive vascular patterns, is essential for precise diagnosis and management, thus preventing overtreatment that could result from a misdiagnosis of malignancy.
